# On the Mechanism of Drug Release from Polysaccharide Hydrogels Cross-Linked with Magnetite Nanoparticles by Applying Alternating Magnetic Fields: the Case of DOXO Delivery

**DOI:** 10.3390/gels1010024

**Published:** 2015-05-20

**Authors:** Marianna Uva, Lorenzo Mencuccini, Andrea Atrei, Claudia Innocenti, Elvira Fantechi, Claudio Sangregorio, Melania Maglio, Milena Fini, Rolando Barbucci

**Affiliations:** 1 Dipartimento di Biotecnologie, Chimica e Farmacia, Università di Siena, 53100 Siena, Italy; E-Mails: marianna.uva@unisi.it (M.U.); lorenzo.mencuccini@unisi.it (L.M.); andrea.atrei@unisi.it (A.A.); 2 Centro Interuniversitario Sistemi Medici Avanzati (CRISMA), 53034 Colle di Val d’Elsa, (Siena), Italy; 3 Dipartimento di Chimica “U. Schiff”, Università di Firenze and INSTM UdR Firenze, 50019 Sesto Fiorentino (Firenze), Italy; E-Mails: claudia.innocenti@unifi.it (C.I.); e.fantechi@gmail.com (E.F.); 4 CNR-ICCOM and INSTM, 50019 Sesto Fiorentino (Firenze), Italy; E-Mail: claudio.sangregorio@iccom.cnr.it; 5 Laboratorio Studi Preclinici e Chirurgici, Istituto Ortopedico Rizzoli, 40136 Bologna, Italy; E-Mails: melania.maglio@ior.it (M.M.); milena.fini@ior.it (M.F.); 6 Laboratorio Biocompatibilità, Innovazioni Tecnologiche e Terapie Avanzate, Istituto Ortopedico Rizzoli, 40136 Bologna, Italy

**Keywords:** Magnetic hydrogels, magnetite nanoparticles, carboxymethylcellulose, FESEM, magnetic properties, DOXO release

## Abstract

The chemical, biological and physical properties of carboxymethylcellulose (CMC) hydrogels with silanized magnetite (Fe_3_O_4_) nanoparticles (NPs) as cross-linker were investigated and compared with the analogous hydrogel obtained by using 1,3-diaminopropane (DAP) as cross-linker. The magnetic hydrogel was characterized from the chemical point of view by FT-IR, whereas the morphology of the hydrogel was investigated by FESEM and STEM. The water uptake and rheological measurements reveal how much the swelling and mechanical properties change when CMC is cross-linked with silanized magnetite NPs instead of with DAP. As far as the biological properties, the hybrid hydrogel neither exerts any adverse effect nor any alteration on the cells. The magnetic hydrogels show magnetic hysteresis at 2.5 K as well as at 300 K. Magnetic measurements show that the saturation magnetization, remanent magnetization and coercive field of the NPs are not influenced significantly by the silanization treatment. The magnetic hydrogel was tested as controlled drug delivery system. The release of DOXO from the hydrogel is significantly enhanced by exposing it to an alternating magnetic field. Under our experimental conditions (2 mT and 40 kHz), no temperature increase of the hydrogel was measured, testifying that the mechanism for the enhancement of drug release under the AMF involves the twisting of the polymeric chains. A static magnetic field (0.5 T) does not influence the drug release from the hydrogel, compared with that without magnetic field.

## 1. Introduction

As therapeutic tools, magnetic nanoparticles (NPs) have been evaluated extensively for local delivery of pharmaceuticals via magnetic drug targeting [1,2,3,4,5,6] and via attachment of high affinity ligands [7,8,9]. Thus, while offering an attractive mean for remotely directing therapeutic agents specifically to a diseased site, the magnetic NPs simultaneously reduce dosage and deleterious side effects associated with non-specific uptake of cytotoxic drugs by healthy tissues [10,11,12,13,14,15,16,17]. NPs have been used to treat tumors in several ways. Specific antibodies can be conjugated to the magnetic NPs to bind selectively to related receptors and thus inhibit tumor growth; magnetic NPs can be used for hyperthermia and also for targeted delivery of antitumor agents adsorbed on their surface [18,19,20,21]. Nevertheless, the small size of NPs induces researchers to utilize large amount of them to get an appreciable quantity of drug in the diseased site, their presence in the organism, especially in large amount, is certainly to be avoided. To overcome this health issue, NPs can be enclosed into the polysaccharide matrix of a hydrogel. Hydrogels represent ideal drug delivery systems since the duration of the release and delivered dose can be controlled. Moreover other intrinsic characteristics of hydrogels are the low cost, excellent biocompatibility, the possibility of chemical functionalization and last but not the least, the possibility to entrap large quantity of drug [22,23]. 

In conclusion, the combination of polymeric hydrogels and magnetic NPs represents one of the most promising approaches for the delivery system, conserving both hydrogel and magnetic properties [24]. There are several methods to obtain magnetic NPs entrapped inside hydrogels: NPs can be directly introduced into the matrix of a preformed hydrogel or they can be added during the gel formation process, or otherwise they can be used as cross-linkers between the polymer chains [25,26,27]. The last method is the strategy that we utilize, which involves the formation of a covalent bond between the polymeric chains (carboxymethylcellulose, CMC) and the conveniently modified surface of the magnetic NPs. The major advantage of this strategy is that the covalent bond avoids the release of NPs from the hydrogel matrix, which could result in toxicity for the host tissue. In a previous work, we used CoFe_2_O_4_ NPs, modified with (3-aminopropyl)-trimethoxysilane (APTMS), as cross-linkers to prepare a CMC hydrogel [28]. The magnetic hybrid hydrogel was tested as a controlled drug release system. The material resulted very sensitive to the application of an external magnetic field. In particular, the release of model drugs was enhanced by the application of an alternating magnetic field (AMF), while it was inhibited by applying a static magnetic field. This observation opened the opportunity of modulation of the drug release by the combination of alternating and static magnetic field in sequence. 

In the present study, magnetite (Fe_3_O_4_) NPs functionalized with APTMS were used as cross-linker for the synthesis of a CMC-based hybrid hydrogel. Indeed magnetite nanoparticles ranging from the nanometer and micrometer scale have been widely applied in recent years in the area of biomedicine such as contrast agent in magnetic resonance imaging [29,30], drug carriers in targeted drug delivery system [31,32] and magnetic fluids in hyperthermia [33,34], and some different iron oxide formulations are already present on the market, after their regular approval by both the FDA and some European countries. Furthermore, the results of a targeted search of clinical trials registered at PubMed and at the US National Library of Medicine point out the use of iron oxide for magnetic resonance imaging (MRI), for magnetic thermoablation of cancer, and for the development of magnetic biopsy.

The hybrid CMC hydrogel with Fe_3_O_4_ NPs was thoroughly characterized from the morphological, chemical and physical point of view. Moreover, the effects of the new magnetic hybrid hydrogel on the proliferation and differentiation of osteoblasts were investigated. Finally, the new hybrid material was tested, under external magnetic fields, as a drug delivery system for Doxorubicin (DOXO), one of the most commonly used anticancer drugs, due to its broad spectrum efficacy against solid tumors, including bone cancer [35]. 

## 2. Results and Discussion

The synthesis of magnetic hybrid hydrogels consists of two important steps, as already described for the analogous CMC-NPs (CoFe_2_O_4_) hydrogel [24]:

a) functionalization of the magnetite (Fe_3_O_4_) NPs with APTMS in order to introduce -NH_2_ groups on the NPs surface (NPs-NH_2_); and
b) binding of CMC polymer to the (Fe_3_O_4_) NPs-NH_2_, via the formation of an amide bond between the CMC carboxylic groups and the -NH_2_ groups on the NPs surface in order to get the hybrid hydrogel.

### 2.1. Characterization and Morphological Analysis of the NPs-NH_2_ and the Hydrogel CMC-NPs

(Fe_3_O_4_) NPs were functionalized with APTMS [24]. The success of silanization was confirmed by FT–IR analysis that shows –NH2 and Si–O characteristic absorption bands at 1600, 1410, 1100 and 1040 cm^−1^, as already reported for NPs-NH_2_ (CoFe_2_O_4_) [36]. The stability of the dispersions of the bare and silanized NPs (Fe_3_O_4_) was investigated by DLS. The size and polydispersity index (PDI) of the aggregates formed by NPs-NH_2_ (Fe_3_O_4_) are smaller than those of the aggregates formed by bare NPs. (Table 1). These results show that the coating with silane molecules is able to reduce the aggregation and produces more stable and more monodisperse dispersions [37,38]. On top of that, the silanization allows a uniform distribution of the cross-linker agents (*i.e.* the NPs-NH_2_) in the reaction mixture necessary for the synthesis of the hybrid CMC-NPs hydrogel. The amount of NH_2_ groups introduced on the Fe_3_O_4_ NP’s surface is determinable by cyclic voltammetry as obtained previously [36]. Considering for Fe_3_O_4_ NP-NH_2_ an average particle size of 30 nm and a magnetite density of 5.17 g·cm^−3^, the number of APTMS molecules bound to the NPs’ surface calculated is 2.4 ± 0.6 × 10^5^.

**Figure 1:**
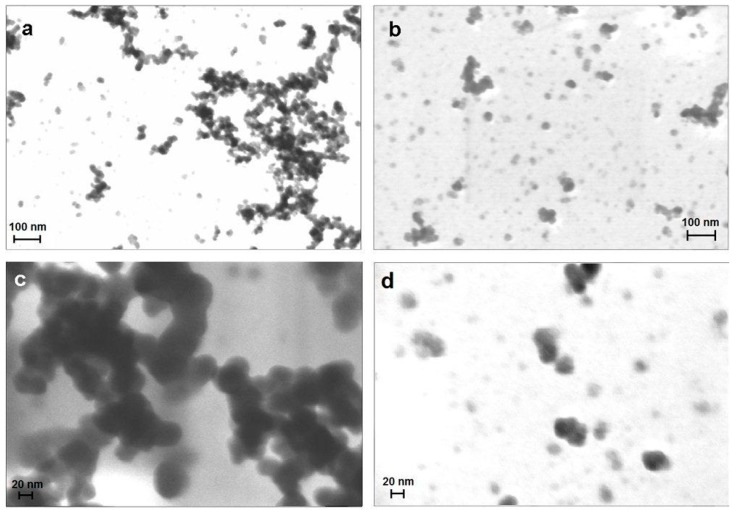
FESEM images of bare nanoparticles (NPs); the bar corresponds to 100 nm (**a**) and 20 nm (**c**); FESEM images of NPs-NH_2_, the bar corresponds to 100 nm (**b**) and 20 nm (**d**).

STEM images of bare and coated NPs confirmed the DLS results (Figure 1). Uncoated Fe_3_O_4_ NPs (Figure 1a,c) show a strong tendency to aggregate and form clusters, even if few isolated single NPs with spherical shape (diameter between 20 and 30 nm) are present. On the contrary, silanized Fe_3_O_4_ NPs-NH_2_ (Figure 1b,d) exhibit a lower tendency to aggregate and isolated single NPs-NH_2_’s, with the same size as the bare NPs (20–30 nm), are apparent.

**Table 1:** Size, polydispersity index and ζ-potential of NPs and NPs-NH_2_ at pH 5.

	Fe_3_O_4_ NPs	Fe_3_O_4_ NPs-NH_2_
**Size (nm)**	2004 ± 207	499 ± 10
**PDI** **ζ-potential**	0.88 ± 0.09 17 ± 2	0.35 ± 0.08 23 ± 1

**Figure 2:**
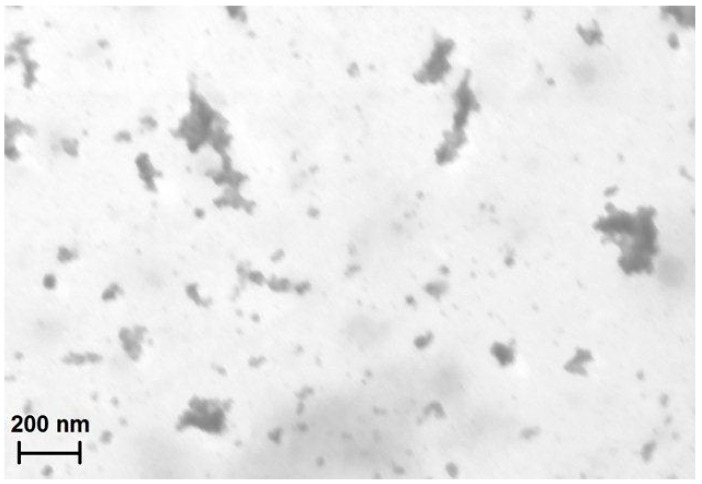
STEM image of the dried hydrogel CMC-NPs. The bar corresponds to 200 nm.

Subsequently, the synthesis of magnetic hydrogels involves the formation of amide bonds between the carboxylic groups of the polysaccharide and the primary amines of the NPs-NH_2_ in the presence of EDC as the activating agent of carboxylic groups and NHS as the proton exchanger [27]. A dark brown hydrogel is formed: the STEM image of the dried CMC-NPs hydrogel shows NP clusters of various sizes (Figure 2).

The advantage of such an approach is that the magnetic nanoparticles are permanently linked in the network, which prevents them from diffusing out of the gel. In addition, the presence of magnetic NPs as cross-linkers in the hybrid hydrogel makes the system susceptible to the influence of magnetic fields: the hydrogel in water is attracted by a magnet and can be moved in all directions. The formation of the amide bond between the silanized nanoparticles and the CMC polymer was ascertained by FTIR analysis of the hydrogel. Both the CMC and the CMC-NPs spectra show the characteristic polysaccharide absorption bands between 1060 and 1400 cm^−1^ [39]; in addition the CMC-NPs hydrogel spectrum shows a band at 1640 cm^−1^, with a shoulder at 1557 cm^−1^, which can be attributed to the C=O amide stretching and amide N–H bending modes, respectively. The absorption band at 1593 cm^−1^ indicates that some carboxylate groups on the polysaccharide chain remain free in the hydrogel. To verify always the presence of the amino groups on the NPs surface, the ninhydrin test was performed on the hydrogel [40]; the negative result of the test means that all the NH_2_ groups, within the sensitivity of the method, bind the carboxylic groups of CMC.

### 2.2. Water Uptake (WU) and Rheological Measurements

**Figure 3:**
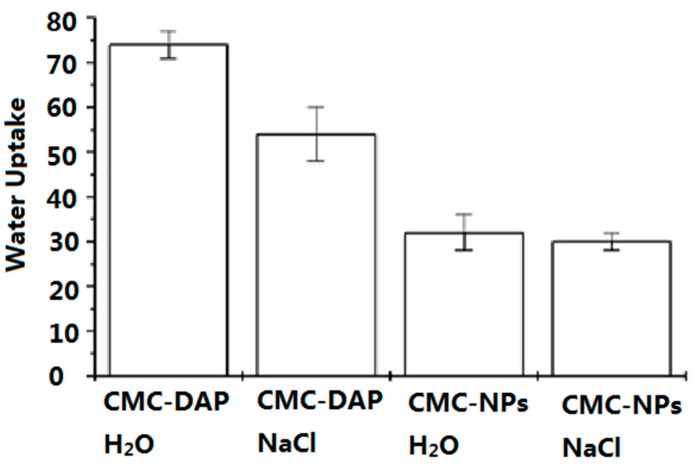
Water uptake (% in weight) for CMC-NPs and CMC-DAP hydrogels in water and in 0.15 M NaCl solution at their maximum swelling degree.

Water uptake values of CMC-NPs and CMC-DAP hydrogels, in water and in 0.15 NaCl solution are shown in Figure 3. Both hydrogels show a high water uptake, due to the hydrophilic nature of the CMC polymer and also to the formation of numerous highly hydrophilic amide groups for the cross-linking reaction. Nevertheless the WU of CMC-NPs (Fe_3_O_4_) is lower than that CMC-DAP hydrogel, a behavior that was also already observed for CMC-NPs (CoFe_2_O_4_) [36]. This confirms that the presence of NPs reduces the ability of the hydrogel to swell because of more tightly linked structures. No significant differences were observed between the WU of CMC-NPs hydrogel in water and that in NaCl 0.15 M. The tight structure of CMC-NPs hydrogel cancels the effect of the ionic strength on the swelling degree.

The rheological properties of the CMC-NPs (Fe_3_O_4_) hydrogel were compared with those of the CMC-DAP hydrogel (Table 2). The dynamic mechanical analysis indicated a “gel-like” behavior, with the G' greater than the G" within the frequency range for both the samples. The presence of NPs in the hydrogel matrix gives rise to higher elastic modulus values for the CMC-NPs hydrogel. On the contrary, no significant difference in the G" values were observed, indicating a very similar viscous behavior [15]. An analogous trend was also observed for the CMC-NPs (CoFe_2_O_4_) hydrogel [28], even if the viscosity character of CMC-NP (Fe_3_O_4_) results are greater than that of CMC-NP(CoFe_2_O_4_). A different degree of cross-link between the CMC-DAP hydrogel and the CMC-NP hydrogel (which were not determined) may have an effect on the observed differences in the WU and rheological properties.

**Table 2:** G' and G" values for CMC-NPs and CMC-DAP hydrogels.

Sample	G' (Pa)	G" (Pa)
CMC-DAP	1025 ± 300	300 ± 90
CMC-NPs (Fe_3_O_4_)	3250 ± 120	258 ± 30

### 2.3. Magnetic Properties

**Figure 4:**
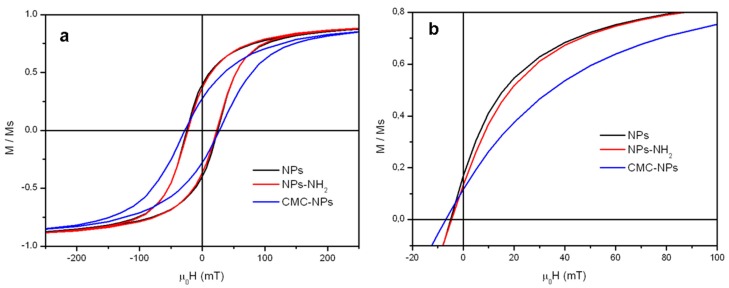
Magnetization curves normalized to the corresponding saturation magnetization MS, reported in Table 3, measured at 2.5 K (**a**) and 300 K (**b**).

The magnetic properties of the NPs-NH_2_ and the dry CMC-NPs hydrogel were investigated and compared with those of the pristine NPs. The low temperature (2.5 K) field dependence of magnetization (Figure 4a) shows open hysteresis loops for the three samples. The values of coercive fields *μ_0_H_C_,* reported in Table 3, are very close for all samples and are consistent with the data reported in the literature for nanosized iron oxides of similar size [41]. On the contrary, the reduced remanent magnetization, *M_R_* = *M_0T_/M_5T_*, clearly decreases for CMC-NPs (*M_R_* = 0.27) if compared to NPs (*M_R_* = 0.39) and NPs-NH_2_ (*M_R_* = 0.37). This behavior can be ascribed to the presence of a larger degree of interparticle interactions, mainly exchange ones [42], in the NPs and the silanized NPs samples, which were measured as a pellet of pressed powder. Indeed, measurements on a diluted solution of silanized NPs, where interactions are expected to be reduced, provide a decreased value close to that observed for CMC-NPs, while keeping the coercive field unchanged.

**Figure 5:**
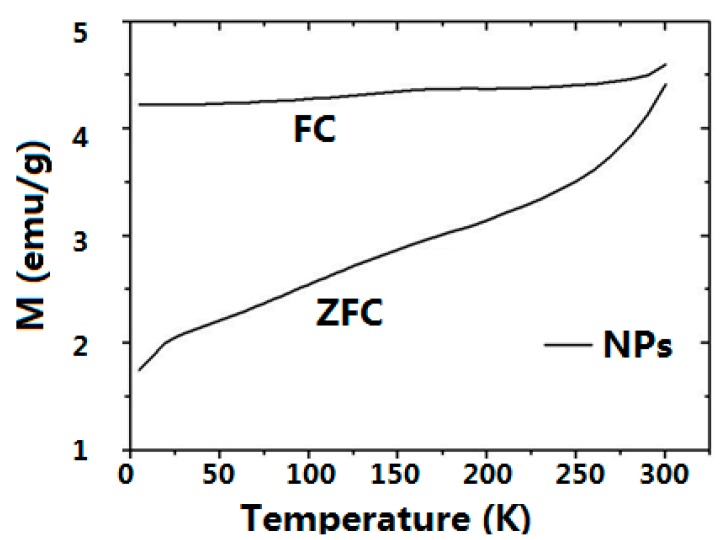
ZFC/FC magnetizations measured for NPs obtained by applying a 5 mT probe field.

The three samples also show a small coercivity and remanence at room temperature (Figure 4b), suggesting that the system is not superparamagnetic at this temperature. This result is coherent with the size of the nanoparticle and is further confirmed by the temperature dependences of the ZFC and FC magnetizations (Figure 5), which show a progressive increase of the ZFC magnetization with temperature without reaching any maximum in the observed temperature range to identify the blocking temperature of the system. As expected, the values of *H_C_* and *M_R_* are lower than those measured at low temperature. The saturation magnetizations, *M_S_*, were estimated by the values recorded at 5 T, since the saturation is almost reached in the high field region of the curve. In Table 3, the *M_S_* values are normalized to the weight of the measured sample, which for NPs corresponds to the effective amount of magnetite content. The slight difference between *M_S_* obtained for NPs and NPs-NH_2_ due to the small amount of silane coating can be neglected, while the lower value reported for the dry CMC-NPs is ascribed to the contribution of the dry hydrogel to the total weight of the sample. The *M_S_* value obtained for NPs and NPs-NH_2_ matches well the one reported in the literature for iron oxide of similar size. The recorded *M_S_* values are indeed *ca*. 10%–15% lower than those of bulk magnetite. The reduction of the saturation magnetization is commonly observed in nanosized magnetic material and can be ascribed to the presence of a dead spin layer on the surface, whose contribution becomes more and more important with size reduction.

**Table 3:** ^a,d^ Coercive field at 2.5 K (a) and 300 K (d); ^b,e^ saturation magnetization at 2.5 K (b) and 300K (e); ^c,f^ reduced remanent magnetization (M_0T_/M_5T_) at 2.5 K (c) and 300K (f). Magnetizations are normalized to the weight of dry samples.

Sample	*_0_Hc* 2.5 K (mT) ^a^	*M_S_* 2.5 K (Am^2^/kg) ^b^	M_R_ 2.5 K ^c^	*_0_Hc* 300 K (mT) ^d^	*M_S_* 300 K (Am^2^/kg) ^e^	M_R_ 300 K ^f^
NPs	24.6	86	0.39	5.0	76	0.17
NPs-NH_2_	23.2	83	0.37	4.6	74	0.13
CMC-NPs hydrogel	27.8	16	0.27	6.8	14	0.12

We measured the temperature variation induced by the application of an AMF to the CMC hydrogel with silanized magnetite NPs as cross-linkers. The measurements were performed on hydrogels swollen in water. An AMF with a frequency of 54 kHz and an intensity of 2.2 mT similar to the frequency and intensity of the AMF used in the drug release experiments (see Section 2.5 and Section 4.2.10) was applied for five minutes. Under these conditions, no temperature increase above 0.1 K was observed. Moreover, when using CMC-DAP hydrogel with Fe_3_O_4_ NPs seeded (not linked to the polymeric chains), no increase of temperature was observed in the same conditions.

In order to complete the characterization of the behavior of CMC-NPs hydrogels and to investigate their capability to release heat under the effect of AMF, the temperature variation of the gel was also measured using higher amplitudes and frequencies within the range of those commonly used for magnetic fluid hyperthermia, A temperature increment of 9 K was measured for the CMC-NP hydrogel after the application for five minutes of an AMF at a frequency of 183 kHz and an intensity of 21.2 mT. At these frequencies and field amplitudes, the NPs inside the CMC gels shows hyperthermic properties, which were not highlighted at lower field parameters due to the non sizeable heat release.

On the basis of the observed data, even though the AMF with the parameters used in the drug release tests does not lead to a macroscopic, measurable temperature increase of the whole sample, we cannot rule out a temperature rise at the surface of the NPs. Indeed, a local heating (tens of K) around iron oxide NPs produced by an AMF was already foreseen theoretically [43] and experimentally measured [44]. It should be noted that, in this latter work, the frequency and intensity of the AMF were higher than those used in our experimental conditions.

### 2.4. Biological Tests

**Figure 6:**
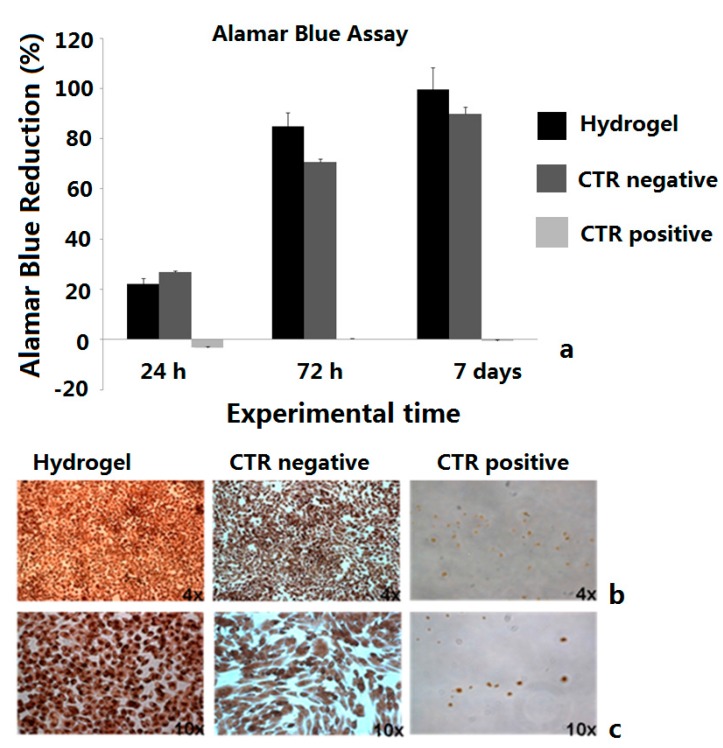
MG63 proliferation (**a**). Neutral red staining of MG63 at different culture conditions after 48 h, magnification 4× (**b**) and 10× (**c**). Tests were performed on the CMC-NP (Fe_3_O_4_) hydrogels.

Alamar Blue assay results showed a proliferative trend, increasing overtime, in cells cultured with the CMC-NP (Fe_3_O_4_) hydrogels, comparable to negative control (Figure 6a).

**Figure 7:**
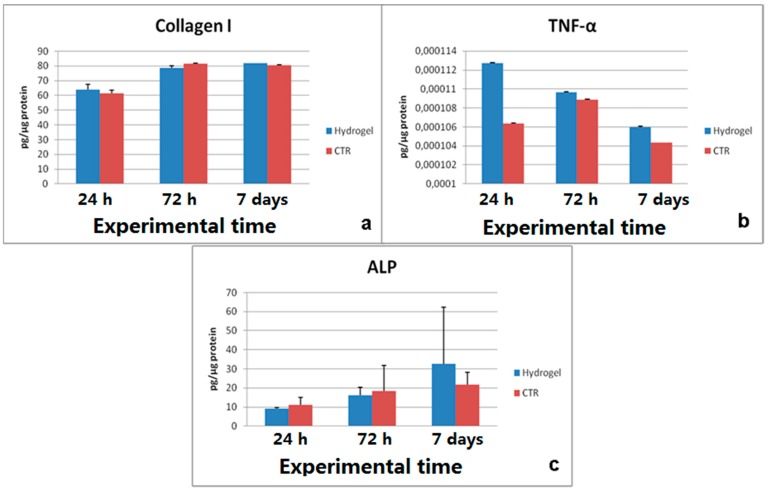
MG63 activity (**a**–**c**) after 24 h, 72 h and 7 days culture. Results are the mean ± SD; no statistical significance was detected between the group with the material and the negative control. Tests were performed on the CMC-NP (Fe_3_O_4_) hydrogels.

Evaluation of LDH release (data not shown) and Neutral red staining confirmed the absence of cytotoxicity in the culture with the material (Figure 6b,c), in which cells appeared strongly colored and with similar morphology of negative control ones. The osteoblastic activity seems to be unaffected, with increasing ALP production, COLL I synthesis and low values of TNFα, indicating the absence of inflammatory process (Figure 7a–c). No statistical significance has been detected between experimental and control group at any experimental time of any test conducted.

### 2.5. Release of Doxorubicin from Drug-Loaded CMC-NPs Hydrogel

**Figure 8:**
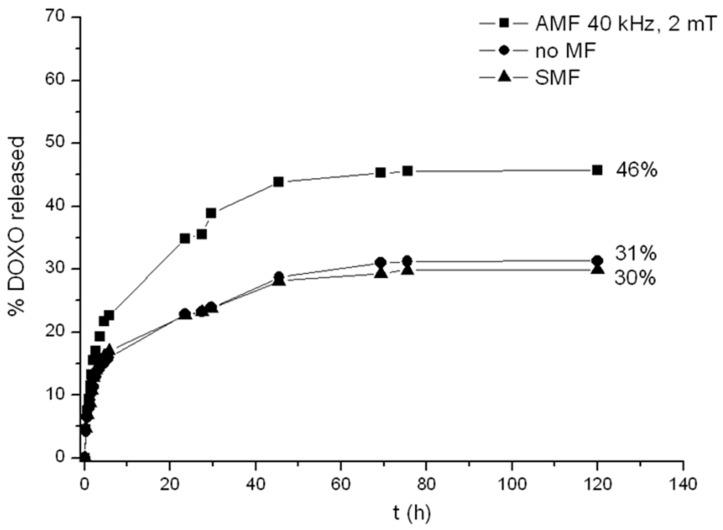
Comparison of the release profiles of DOXO from CMC-NPs in NaCl 0.15 M in the absence of MF (circles), in the presence of AMF (squares), and with SMF (triangles).

*In vitro* drug release of DOXO loaded into CMC-NPs (Fe_3_O_4_) magnetic hydrogels with and without alternating (40 kHz, 2 mT) and static magnetic fields (0.5 T) was carried out in 0.15 M NaCl, at RT. The release experiments were performed for CMC-NPs hydrogels loaded with a DOXO concentration of 66 ± 3 µg DOXO per mg of dried hydrogel. However, DOXO loading efficiency of the CMC-NPs hydrogel can be as high as 950 ± 50 µg per mg of dried hydrogel. This loading capacity is significantly higher than the values previously reported for other systems like lipid, micelles, and polymeric matrices [45,46,47]. As shown in Figure 8, CMC-NPs hydrogel always exhibits a sustained release of DOXO during the first 48 h for all three samples (in the presence of AMF, without any magnetic field and in the presence of SMF). Without magnetic field, the total amount of DOXO released was quite low (31%), in the presence of SMF (30%), and with AMF (46%).

First, let us consider the effect of the SMF on the drug release. No changes, either in the drug release kinetics or in the total amount of DOXO released, were observed with DOXO-loaded CMC-NPs (Fe_3_O_4_) hydrogel under the SMF in comparison to the values obtained without magnetic field. On the contrary, a reduction of a dye (methylene blue) release had been observed for the CMC-NPs (CoFe_2_O_4_) hydrogel when the SMF was applied [28]. In the latter system, an elongation of the hydrogel subjected to the SMF was observed and the reduction of drug release was explained in terms of a reduction of the hydrogel porosity when elongated by the magnets [28]. No macroscopic elongation of the CMC-NPs (Fe_3_O_4_) hydrogel was observed when a SMF is applied. Even if the structure complexity of the DOXO molecule in comparison of that of Methylene blue dye and, consequently, the interaction with the CMC-NP hydrogel does no have to be completely excluded; this different behavior is likely due to a bigger aggregation of the Fe_3_O_4_ nanoparticles and to the larger number of NP clusters in the hydrogel, as clearly showed by FESEM image. Nevertheless a stronger static magnetic field than that utilized (0.5 T) may generate the elongation of the hydrogel CMC-NP(Fe_3_O_4_) .The lower swelling degree of the CMC-NPs (Fe_3_O_4_) hydrogel, in comparison to that of the CMC-NPs (CoFe_2_O_4_) hydrogel, indicates a tougher structure for the former (less capable to deform its shape), even if the amount of magnetic nanoparticles was almost the same for both samples.

**Figure 9:**
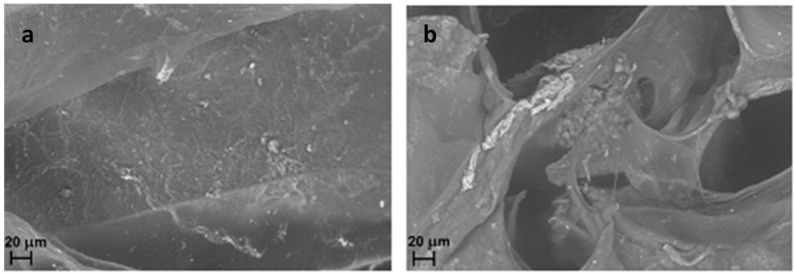
FESEM images of drug-loaded CMC-NPs hydrogel without application of magnetic field (**a**) and with application of AMF (**b**).

**Figure 10:**
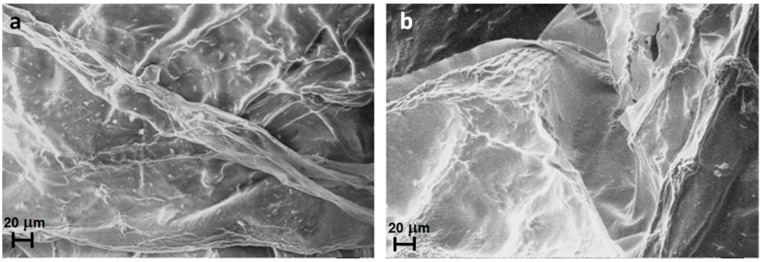
FESEM images of CMC-DAP hydrogel with free Fe_3_O_4_ NPs inside before (**a**) and after (**b**) the application of the AMF.

When the AMF is applied to CMC-NPs (Fe_3_O_4_), an increase of 16% in the total amount of drug released by the hydrogel compared to that without magnetic field was observed. This effect can be explained on the basis of the following hypothesis. Clusters of NPs of different sizes are expected to follow the oscillation of the magnetic field in different manners. The smaller aggregates should follow the change of polarity more easily than the bigger ones because the magnitude of torque exerted by the field depends on the dimensions of the NPs aggregates [48,49]. The different rotations of the aggregates connected to each other by the polymer strands induce a twisting of the polymeric chains with the consequent local breaking of the structure. FESEM images in Figure 9 show the morphology of drug-loaded CMC-NPs hydrogel before (Figure 10a) and after (Figure 10b) the application of AMF. The hydrogel surface appears smooth, but the AMF tears it up. The release of DOXO molecules is made easier by such a broken hydrogel [28]. Moreover, we performed tests investigating the DOXO release from a purposely-synthesized CMC-DAP hydrogel with Fe_3_O_4_ NPs only seeded. No difference in the release kinetics and in the total amount of drug released with or without the application of AMF was observed. Moreover, FESEM images (Figure 10) show no variation in the morphology of the CMC-DAP hydrogel with embedded (Fe_3_O_4_) NPs after the application of the AMF. This is further evidence that the effect of the AMF on the hydrogel morphology, and thus on the drug release, occurs only when the NPs are covalently bonded to the polymer network.

## 3. Conclusions

In this paper, we investigated hydrogels with magnetic (Fe_3_O_4_) NPs used as cross-linkers of polysaccharide chains. This hybrid system combines the properties of hydrogels with those of the magnetic NPs. The hydrogel then allows uptake of significant amounts of drug (the amount of the antitumor DOXO drug loaded reached 950 microgram/(mg of CMC-NP dry hydrogel)) while the nature of the polysaccharide makes it extremely biocompatible. No toxicity for osteoblast was in fact observed over seven days and the presence of the material did not interfere with the normal activity of cells, instead it enhanced the proliferation of the cells with respect to cells cultured in the absence of the hydrogel.

The drug release from the hydrogel can be significantly enhanced by using an AMF. Under our experimental conditions (2 mT and 40 kHz), no temperature increase of the hydrogel was measured. Besides, the magnetite NPs in the CMC hydrogel exhibit a hysteretic behavior at room temperature, thus absorbing power from the AMF at any frequency and inducing torques on the NPs, which are transmitted to the polymer strands to which the NPs are covalently bound. This effect can only occur when the NPs are the nodes of the polymeric network. The formation of cracks and fissures in the hydrogel matrix makes the release of DOXO easier. On the contrary, the application of a static magnetic field (0.5 T) does not affect the release properties of the CMC-NP (Fe_3_O_4_) hydrogel. This might be due to the partial strong aggregation of the nanoparticles in the hydrogel, as revealed by FESEM image. Nevertheless, this novel biomaterial seems to show suitable properties to be considered as a good drug controlled release system by AMF.

## 4. Materials and methods

### 4.1. Materials

The sodium salt of CMC, average MW 700 kDa, with a degree of carboxymethylation equal to 0.86 and magnetite NPs were provided by Sigma-Aldrich (Saint Louis, MO, USA). According to the manufacturer’s specifications, the nominal diameter of the primary Fe_3_O_4_ NPs is about 30 nm. The silane coupling agent APTMS, *N*-hydroxysuccinimide (NHS), *N*-(3-dimethylaminopropyl)-*N*-ethylcarbodiimide hydrochloride (EDC), 1,3-diaminopropane (DAP), DOXO hydrochloride, and all the reagents were purchased from Sigma-Aldrich.

### 4.2. Methods

#### 4.2.1. Functionalization of Fe_3_O_4_ NPs

The surface of magnetite NPs was functionalized using APTMS, according to a procedure already described [24]. Briefly, 1.68 mL of APTMS were added to a suspension containing 0.5 g of NPs in a 95%/5% *v*/*v* ethanol/water mixture at pH 5, corresponding to a molar ratio of 4.5:1 with respect to the moles of Fe_3_O_4_ present in the NPs suspension. The functionalized NPs (NPs-NH_2_) were precipitated by centrifugation at 6000 rpm and washed with ethanol before being dried in an oven at 60 °C overnight.

#### 4.2.2. Synthesis of the Hybrid Hydrogel

First, 0.5 g CMC were dissolved in deionized water. NHS and EDC were added to this solution at a molar ratio of 1 with respect to the moles of the carboxyl groups of the polysaccharide. Then, 0.25 g of NPs-NH2 were added to the mixture as cross-linker and the pH was adjusted to 7 with 0.1 M NaOH. Once formed, the hydrogel was purified by washing in water until no traces of EDC or NHS were revealed by UV spectroscopy. The amount of Fe_3_O_4_ present in the CMC-NPs hydrogel samples (*ca.* 40 weight % with respect to the polymer) was measured by spectrophotometry of the complex between o-phenanthroline and Fe^2+^ after dissolving the hydrogels. Furthermore a hydrogel of CMC was prepared using DAP as cross-linker (referred to as CMC-DAP), following the previously reported chemical route [50], and was investigated as control sample in the mechanical and chemical measurements.

#### 4.2.3. FT-IR and UV-Visible Spectroscopy

Fourier transform infrared (FT-IR) spectroscopy measurements were carried out at room temperature on a Bio-Rad FTS 6000 FT-IR spectrometer (Bio-Rad, Hercules, CA, USA) equipped with an attenuated total reflection (ATR) accessory (Ge crystal, 45° angle of incidence) and a mercury-cadmium-telluride detector. All spectra were recorded in the wavenumber range of 750–4000 cm^−1^. The hydrogel samples were lyophilized and placed on the ATR crystal. Spectra were averaged over 128 scans at a resolution of 4 cm^−1^ with baseline correction and smoothing, using WIN-IR PRO version 2.6 software.

UV spectra were acquired at room temperature on a Perkin Elmer Lambda 650 UV/Vis Spectrophotometer (Perkin Elmer, Waltham, MA, USA), using a 1 cm cell path length for data between 300 and 600 nm, with a 1 nm sampling interval.

#### 4.2.4. Dynamic Light Scattering (DLS)

The size distribution of bare and coated magnetite NPs was investigated by dynamic light scattering (DLS), using a Zetasizer NanoZS90 instrument (Malvern, Worcestershire, UK). All measurements were carried out at 24 °C. Both bare and coated NPs were dispersed in 1 mM NaCl at pH 5. The cumulant method was applied to data in order to determine the hydrodynamic diameters of the NPs.

#### 4.2.5. FESEM and STEM

FESEM (Field Emission Scanning Electron Microscopy) and STEM (Scanning Trasmission Electron Microscopy) were performed using a ZEISS FESEM SIGMA VP. The FESEM images were recorded using accelerating voltages in a range of 10–20 kV. Hydrated hydrogels CMC-NPs were observed under low vacuum by using the variable-pressure secondary electron detector (VPSE) or the backscattered electron detector (BSE). The STEM images of dried bare and functionalized magnetic NPs and dried CMC-NPs, were performed at an accelerating voltage of 20 kV and in bright field imaging mode.

#### 4.2.6. Water Uptake

The water uptake (WU) was determined gravimetrically following the previously reported procedure [36]. Freeze dried samples (CMC-NPs and CMC-DAP) were soaked in water and in 0.15 M NaCl solution at room temperature and stored for more than 24 h, until they reached a swelling equilibrium. The WU was calculated using the following equation:

(1) WU = (Ws − Wd)/Wd

where Ws is the weight of the swollen hydrogel at equilibrium and Wd is the initial weight of the dried hydrogel.

#### 4.2.7. Rheological Measurements

Stress sweep tests were conducted on both CMC-NPs and CMC-DAP samples at their maximum swollen state using a strain-controlled TA-Instruments AR2000 Rheometer in the parallel plate configuration, at a controlled temperature of 25 ± 1 °C and at a frequency of 1 Hz. During the test, the sample is subjected to an increasing or decreasing stress, while frequency and temperature are maintained constant. The test gives the values of the storage modulus (G') and the loss modulus (G"). The G' values provide information about the energy stored in the material during deformation stress, while G" describes its viscous character.

#### 4.2.8. Magnetic Properties

The magnetic properties of NPs, NPs-NH_2_ and CMC-NPs samples were investigated using a Quantum Design MPMS SQUID magnetometer, operating in the 1.8–350 K temperature range, with an applied field up to 5 T. Measurements were performed on powder or lyophilized samples, hosted in a Teflon sample holder. Zero Field Cooled-Field Cooled (ZFC/FC) curves were obtained by measuring the temperature dependence of the magnetization applying a probe magnetic field (5 mT) after cooling the sample in the presence (FC) or in the absence (ZFC) of the field.

The hyperthermic properties of CMC-NPs were investigated by means of calorimetric measurements. The temperature of an aliquot of hydrated hydrogel was recorded during the exposition to an AMF. The experimental set-up, based on a 6 kW Fives Celes^®^ power supply, two water-cooled induction coils and a series of variable capacitors, is able to produce an alternate magnetic field with variable frequency (50–400 kHz) and field amplitude (up to 23.8 mT). In this work, the frequency of 54 kHz and field amplitude of 2.2 mT were chosen as the closest to the ones used in the drug release experiments and the field amplitude was varied between 1.8 and 12.6 kA/m. The temperature of the sample was recorded by an optical fiber thermometer connected to a digital temperature recorder during all the exposition to an alternate magnetic field. Since the measurements are carried in non-adiabatic conditions, the Δ*T/*Δ*t* values were extrapolated from the initial slope (*t*→0) of the temperature kinetic curves.

#### 4.2.9. Biological Tests 

CMC-NPs hydrogel, in parts of 5 mg each, was sterilized by immersion in 70% ethanol for 30 min and washed with PBS plus 2% penicillin/streptomycin. After overnight UV irradiation (230 V at 50 Hz) in this solution, materials were pre-wetted in complete culture medium.

MG-63 human osteoblast-like cells, cultured in DMEM medium (Sigma Aldrich, St. Louis, MO, USA) with 10% FCS (Lonza, Visp, Switzerland) and antibiotics (100 U·mL^−1^ penicillin, 100 µg·mL^−1^ streptomycin), were plated in a 24-well plate (5 × 10^4^ cells/mL) with osteoblast activating medium (DMEM + 10^−2^ M-glicerophosphate + 50 µg·mL^–1^ ascorbic acid). After seeding, hydrogels were placed in the wells with cells. Cells cultured in differentiating medium alone and cells cultured in differentiating medium plus 0.5% phenol were used as negative and positive controls, respectively. Cultures were maintained at 37 °C, 5% CO_2_ and assessment were performed in triplicate at different experimental time (24 h, 72 h, 7 days).

Cell proliferation and viability were assessed by Alamar Blue dye test (Serotec, Kidlington, UK). After incubation of the reagent with samples (1:10 *v*/*v*) for 4 h at 37 °C and 5% CO_2_, the absorbance was read at 570 and 600 nm wavelengths using a MicroPlate reader (BioRad, Hercules, CA, USA) and elaborated following manufacturer’s instruction. Lactate dehydrogenase assay (Roche Diagnostics, GmbH, Mannheim, Germany) for cytotoxicity was performed at 24 and 48 h, incubating supernatants with reagent (1:1 ratio) for 30 min at RT in the dark. The absorbance was measured at 490/655 nm and the cytotoxicity percentage was calculated. Neutral red staining (Sigma Aldrich, St. Louis, MO, USA) for cell morphology and viability was performed at 48 h, adding the reagent dye at each culture well (1:10 ratio) and incubating for 90 min at 37 °C. Cells images were taken using a standard light microscope (Olympus IX71, Olympus Italia Srl, Milano, Italy) equipped with a digital camera (XCell, Olympus Italia Srl, Milano, Italy) at 4× and 10× magnification. Supernatants from experimental cultures and negative controls were collected, stored at −80 °C and assayed for Collagen I (COLL I, Enzyme-linked Immunosorbent Assay Kit Uscn, Life Science Inc., Wuhan , China), Tumor necrosis factor alpha (TNF-α, Instant ELISA EBIOSCIENCE, San Diego, CA, USA) and Alkaline Phosphatase (ALP, Enzyme-linked Immunosorbent Assay Kit Uscn, Life Science Inc., Wuhan, China). Concentrations were normalized by Total Protein content (TP, Total Protein micro-Lowry kit, Sigma Aldrich, St. Louis, MO, USA) to take into account the differences in cell growth.

Statistical evaluation of data was performed using the SPSS Inc. v.12 software. Data are reported as mean ± standard deviations (SD) at a significance level of *p* < 0.05. Mann-Whitney U was performed to detect significant differences between a group with experimental material and another one of cells cultured in standard condition within each experimental time.

#### 4.2.10. Drug Loading and Release

CMC-NPs hydrogel samples were loaded by DOXO from a freshly prepared aqueous solution of the drug. A weighted amount of freeze-dried hydrogel (2 mg) were soaked in 10 mL of 0.018 mM DOXO solution for 7 days under gentle mechanical stirring in order to allow the hydrogel to reach the maximum swelling degree. The amount of DOXO loaded into the samples was estimated by the difference between the initial feeding amount of DOXO and the residual amount of DOXO in the solution after hydrogel soaking (both these quantities were measure by UV-Visible spectroscopy). The hydrogel samples were then removed from the excess of water and dried at room temperature. The dried DOXO-loaded hydrogels were then resuspended in 3 mL of 0.15 M NaCl solution.

The release of DOXO was investigated at room temperature, in the absence of magnetic field, under the effect of an AMF (magnetic induction of 2 mT and frequency of 40 kHz) and under the effect of a static magnetic field (SMF) with magnetic induction of 0.5 T. The AMF was generated by placing the sample between two copper solenoids connected by two ferrite bars. The SMF was instead generated placing the samples at a distance of 1 mm from two 75 mm long permanent magnets, each composed of 15 magnets, 5 mm long. The measurements were carried out in the dark, in order to prevent the degradation of DOXO under illumination [36]. No degradation was observed up to 120 h. The absorbance at 497 nm was used to determine the concentration of DOXO released in the solution as a function of time.
